# FtsZ-mediated fission of a cuboid bacterial symbiont

**DOI:** 10.1016/j.isci.2021.103552

**Published:** 2021-12-02

**Authors:** Philipp M. Weber, Gabriela F. Paredes, Tobias Viehboeck, Nika Pende, Jean-Marie Volland, Olivier Gros, Michael VanNieuwenhze, Jörg Ott, Silvia Bulgheresi

**Affiliations:** 1 Department of Functional and Evolutionary Ecology, Environmental Cell Biology Group, University of Vienna, Djerassiplatz 1, 1030 Vienna, Austria; 2 Evolutionary Biology of the Microbial Cell Unit, Department of Microbiology, Institut Pasteur, 25-28 Rue du Dr Roux, 75015 Paris, France; 3 Department of Energy Joint Genome Institute, Lawrence Berkeley National Laboratory, Berkeley, CA 94720, USA; 4 LRC Systems, Menlo Park, CA 94025, USA; 5 C3MAG, UFR Des Sciences Exactes Et Naturelles, Université Des Antilles, BP 592, 97159 Pointe-à-Pitre, Guadeloupe, France; 6 Indiana University, Bloomington, IN 47405, USA; 7 Division of Microbial Ecology, Center for Microbiology and Environmental Systems Science, University of Vienna, Djerassiplatz 1, 1030 Vienna, Austria; 8 Department of Functional and Evolutionary Ecology, Limnology and Bio-Oceanography Unit, University of Vienna, Djerassiplatz 1, 1030 Vienna, Austria

**Keywords:** Microbial symbiosis, Bacterial cell shape, Bacterial cell division

## Abstract

Less than a handful of cuboid and squared cells have been described in nature, which makes them a rarity. Here, we show how *Candidatus* Thiosymbion cuboideus, a cube-like gammaproteobacterium, reproduces on the surface of marine free-living nematodes. Immunostaining of symbiont cells with an anti-fimbriae antibody revealed that they are host-polarized, as these appendages exclusively localized at the host-proximal (animal-attached) pole. Moreover, by applying a fluorescently labeled metabolic probe to track new cell wall insertion *in vivo*, we observed that the host-attached pole started septation before the distal one. Similarly, *Ca.* T. cuboideus cells immunostained with an anti-FtsZ antibody revealed a proximal-to-distal localization pattern of this tubulin homolog. Although FtsZ has been shown to arrange into squares in synthetically remodeled cuboid cells, here we show that FtsZ may also mediate the division of naturally occurring ones. This implies that, even in natural settings, membrane roundness is not required for FtsZ function.

## Highlights

• *Ca.* T. cuboideus cells are cuboid
• Septation is host oriented in *Ca.* T. cuboideus
• FtsZ localization pattern recapitulates that of new PG insertion
• FtsZ polymerizes into either straight or sharp-cornered filaments

## Graphical abstract

**Figure:**
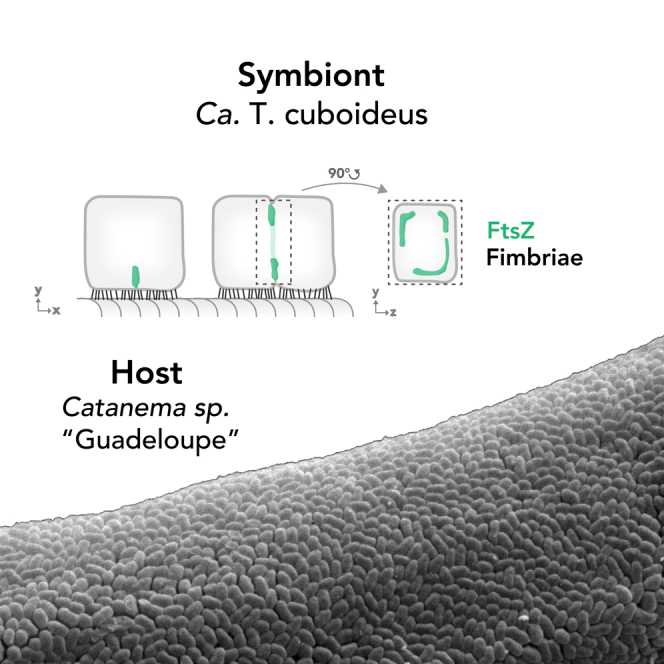

## Introduction

Prokaryotic cells have evolved an enormous diversity of cell shapes and sizes. Although most model bacteria are spheres or rod-like, recently more uncommon shapes, including corkscrews, crescents, or stars, are receiving increasing attention (Caccamo and Brun, 2017; Kysela et al., 2016). However, no square or cuboid bacteria and only two square-shaped archaea have been described so far, *Haloquadratum walsbyi* (Walsby, 1980) and *Haloarcula quadrata* (Oren, 1999).

In bacteria, the rigid peptidoglycan (PG) layer of the cell envelope provides mechanical strength and determines the cell shape. By investigating the molecular mechanisms of PG synthesis, substantial progress has been made in understanding the morphogenesis of model rods such as *Escherichia coli*. Here, the task of directing the PG synthesis machinery is split between the actin homolog MreB and the tubulin homolog FtsZ. Although short MreB filaments are spiraling along the envelope to elongate the cell corpus, FtsZ polymerizes exclusively at the septal plane determining the end of the cell cycle (McQuillen and Xiao, 2020; Shi et al., 2018). In the case of model rods and cocci, FtsZ polymerizes into a discontinuous ring-like structure at septation onset or, at least, during its last step (Eswara and Ramamurthi, 2017). However, in synthetically remodeled cells, fluorescently tagged FtsZ could polymerize into other shapes (including squares), while displaying the usual dynamics (Söderström et al., 2018).

Here, we investigated the reproduction mode of *Candidatus* Thiosymbion cuboideus, a cube-like sulfur-oxidizing gammaproteobacterium, exclusively found attached to the cuticle of marine free-living nematodes (*Stilbonematinae*). We discovered that in this ectosymbiont both FtsZ and newly synthesized peptidoglycan (PG) localize at the septum in a proximal-to-distal fashion, implying that symbiont growth starts at the host-attached pole and that the tubulin homolog may mediate septal PG insertion. We conclude that membrane roundness is not required for FtsZ-based division in natural settings.

## Results

### The ectosymbiont of the marine nematode *Catanema sp*. “Guadeloupe” belongs to the candidate genus *Candidatus* Thiosymbion and bears the *ftsZ* gene

To phylogenetically place the symbiont of the nematode *Catanema sp*. “Guadeloupe” and to characterize its metabolic potential, we dissociated it from its host, extracted its genomic DNA (gDNA) and sequenced it. We estimated the *Ca*. T. cuboideus genome to be 96.91% complete and 5.0 Mb in size (Table S1). A 16S rRNA gene-based phylogenetic tree (Figure S1, Table S2) shows that *Ca*. T. cuboideus clusters together with other *Candidatus* Thiosymbion bacteria that coat *Stilbonematinae*. Furthermore, *Ca*. T. cuboideus possesses the complete *fts* operon, including an *ftsZ* gene whose product is 94% identical to that of the longitudinally dividing *Ca*. T. oneisti and 75% identical to that of the model rod-shaped bacterium *Escherichia coli* (Figures S2A, S2B and Tables S4 and S5).

We conclude that *Ca*. T. cuboideus belongs to the *Candidatus* genus Thiosymbion and that FtsZ may mediate its division.

### *Ca*. T. cuboideus cells are cuboid

**Figure 1:**
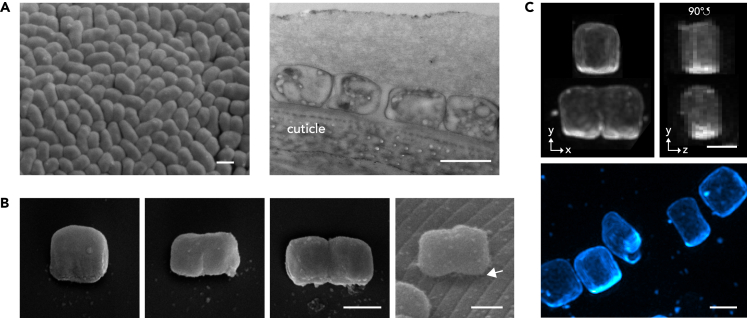
*Ca*. T. cuboideus cells are cuboid (A) A scanning electron micrograph (SEM) showing the top view of the bacterial coat (left) and a transmission electron micrograph (TEM) showing the bacterial coat overlying a transverse section of the nematode (right). (B) SEM images display three representative *Ca*. T. cuboideus cells arranged from the youngest to the oldest (three leftmost panels) and a cell that is attached to the worm cuticle (rightmost panel). Arrowhead points at filamentous structures. (C) 3D-structured illumination microscopy (SIM) images of cells stained with fluorescent wheat germ agglutinin. The top row shows a non-dividing and a dividing cell in front view (yx view, left) and 90° shifted side view (yz view, right; a black rectangle has been placed in the lower right corner to cover a neighboring cell for clarity), and bottom panel shows multiple *Ca*. T. cuboideus cells in different stages of the cell cycle (cyan). Scale bars (A–C) correspond to 1 μm. See also Figures S1 and S3 and Tables S1 and S2.

**Figure 2:**
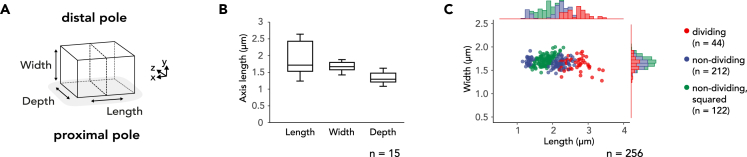
Morphometry of *Ca.* T. cuboideus (A) Schematic representation axes of a *Ca*. T. cuboideus cell indicating its three axes, the proximal (nematode-attached) pole, and the distal (free) cell pole. Length is defined as the axis parallel to the host surface and perpendicular to the division plane, Width as perpendicular to the host surface, and Depth as parallel to the host surface and the division plane. (B) Boxplot shows the Length, Width, and Depth of 15 cells imaged with 3D SIM microscopy. Box is the interquartile range (IQR), where the lower edge is 25^th^ percentile (first quartile [Q1]) and the upper edge the 75^th^ percentile (third quartile [Q3]). Whiskers show the range between the lowest value (Min) and the highest value (Max). Line inside each box indicates the median. (C) Scatterplot shows the axes measurements of 256 *Ca*. T. cuboideus cells, grouped into the categories dividing (red, n = 44), non-dividing (blue, n = 212), and non-dividing, squared (green, n = 122). In non-dividing cells Length and Depth are undiscernible. See also Table S3.

*Ca*. T. cuboideus cells form a continuous monolayer on the cuticle of its marine nematode host *Catanema sp*. “Guadeloupe” (Figure 1A). Henceforth, we will refer to the sides of the symbiont, which are parallel to the nematode surface as poles, namely, the proximal (host-attached) and the distal (free) pole. We define the cell length as the axis parallel to the host surface and perpendicular to the division plane. Three-dimensional (3D) structured illumination microscopy based morphometric analyses revealed both non-dividing and dividing cells grow along their length (Ø = 1.89 ± 0.46 μm), whereas their width (Ø = 1.68 ± 0.14 μm) and depth (Ø = 1.32 ± 0.17 μm) vary only slightly (n = 15, Figures 1C, 2A and 2B). Furthermore, when we measured 256 cells in 2D, we found 58% of the non-dividing cells to be squared (i.e., axes length difference is less than 15%, n = 212; Figure 2C; Table S3).

All in all, based on 3D and 2D morphometric analyses, *Ca*. T. cuboideus cells are cuboid.

### Polarity and asynchronous septation of *Ca*. T. cuboideus

**Figure 3:**
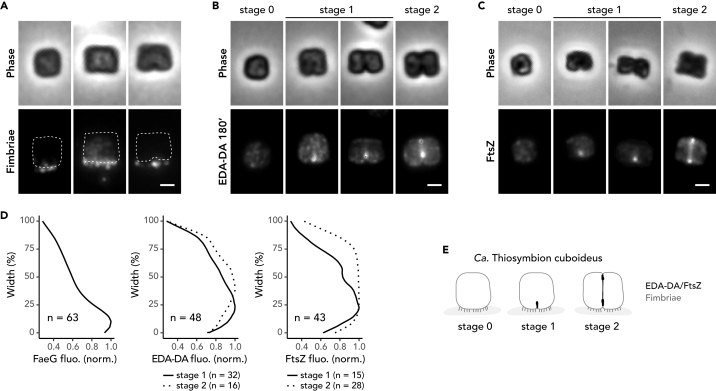
*Ca.* T. cuboideus cells localize FtsZ at their septum (A–C) (A) Three representative cells immunostained with an anti-fimbriae antibody (B) and four cells with a PG metabolic probe EDA-DA, or (C) an anti-FtsZ antibody. (A-C) Phase contrast image (upper panels) and corresponding fluorescence images are shown (lower panels). White dotted cell outline is the cell shape deduced from phase contrast images. Scale bars correspond to 1 µm. (D) Quantitative analyses of the different fluorescence patterns corresponding to the different cell cycle stages. Plots show the normalized fluorescence emitted by *Ca*. T. cuboideus cells (a.u.) stained with an anti-fimbriae antibody (left plot; n = 63), a PG metabolic probe EDA-DA (middle plot; n = 48), or an anti-FtsZ antibody (right plot; n = 43) plotted against their cell width (%). In the middle and right plots cells were split into early (stage 1, full line) or late (stage 2, dotted line) septation stages. (E) Model of *Ca*. T. cuboideus growth and division for non-dividing (stage 0) and dividing (stage1 and 2) cells. See also Figures S2 and S3 and Tables S4 and S5.

To confirm the nature of the filamentous structures observed by scanning electron microscopy (SEM) that are situated between the symbiont proximal cell pole and the nematode cuticle (Figure 1B, right panel and Figure S3B), we immunostained dissociated *Ca*. T. cuboideus cells with an anti-fimbriae antibody. The localization of the epifluorescence signal to one cell side, as previously described for *Ca.* Thiosymbion hypermnestrae (Leisch et al., 2016), indicated that the proximal filamentous structures displayed by *Ca.* T. cuboideus were indeed fimbriae (Figures 3A and 3D left plot, Figure S3C). To track the growth of the cube-like symbiont, we incubated it *in vivo* (i.e., as it was still attached to its nematode host), with a clickable bio-orthogonal PG precursor D-amino acid dipeptide ethynyl-D-alanyl-D-alanine (EDA-DA). We found that the cell walls of symbionts that were incubated in EDA-DA for 3 h were completely stained and that, additionally, dividing cells fluoresced at the nascent septum (Figures 3B, S3A, and S3C). Cells with no visible invagination showed a weak and disperse fluorescence signal (stage 0). In cells that showed an indentation of the proximal pole (early septation stage or stage 1), we detected incorporation of new PG at the proximal pole only. In cells displaying indentations at both poles (later septation stage or stage 2), however, we detected both proximal and distal EDA-DA incorporation (Figures 3B and 3D middle plot; Figure S3C).

We conclude that *Ca*. T. cuboideus cells are host-polarized and that septal growth is asynchronous, starting at the proximal pole, followed by the distal pole.

### FtsZ localization pattern recapitulates that of new PG insertion

To determine whether FtsZ localization would be consistent with septal PG insertion in cuboid bacteria, we immunostained dissociated symbionts with an anti-FtsZ antibody. We found that the FtsZ localization pattern resembled the pattern of insertion of newly inserted septal PG. Indeed, in non-dividing cells a weak FtsZ signal was homogeneously distributed throughout the cell (Figure 3C, leftmost cell). Furthermore, at early septation stages, cells displayed a proximal focus of fluorescence (Figure 3C, two middle cells, and continuous line in Figure 3D) and finally, at later septation stages, they displayed two, one at the proximal and one at the distal pole, with a weaker FtsZ signal in correspondence of the nascent septum, i.e., between the two foci (Figure 3C, rightmost cell, and dashed line in Figures 3D and 3E).

In conclusion, the FtsZ localization pattern suggests that this tubulin homolog may mediate septal PG insertion in cuboid cells.

### *Ca*. T. cuboideus FtsZ polymerizes into either straight or sharp-cornered filaments

**Figure 4:**
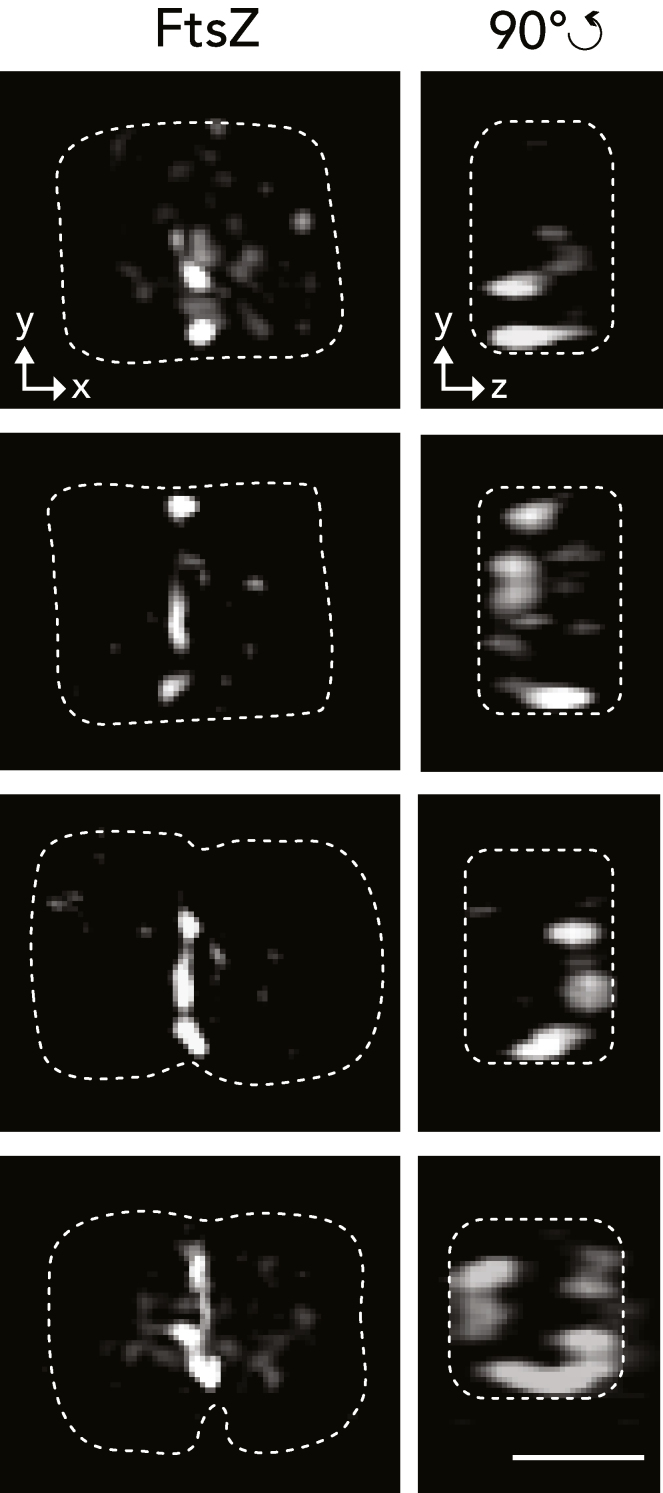
*Ca*. T. cuboideus FtsZ forms either foci or straight or sharp-cornered filaments 3D SIM images of cells immunostained with an anti-FtsZ antibody. No membrane indentations appear in the two top cells, whereas the two bottom cells are invaginated. The left column shows the front view (yx) and the right column a 90° shifted side (yz) view. Dotted lines represent the cell outline of the respective cells (left panels) or the shape of the septum (right panels), deducted from the shape of the increased fluorescence background signal. Scale bar corresponds to 1 μm. See also Figure S2 and Table S5.

To gain a better resolution and a 3D image of the FtsZ localization pattern of immunostained *Ca*. T. cuboideus cells, we performed 3D SIM microscopy. We detected different types of FtsZ arrangement at the septation plane, ranging from foci to straight or sharp-cornered filaments (Figure 4, Videos S1, S2, S3, and S4). Important, the latter arrangements were the largest, as neither continuous FtsZ rings nor squares were observed.

Video S1. 3D reconstruction of fluorescent FtsZ filaments imaged with a 3D SIM microscope of the first cell shown in Figure 2, related to Figure 2

Video S2. 3D reconstruction of fluorescent FtsZ filaments imaged with a 3D SIM microscope of the second cell shown in Figure 2, related to Figure 2

Video S3. 3D reconstruction of fluorescent FtsZ filaments imaged with a 3D SIM microscope of the third cell shown in Figure 2, related to Figure 2

Video S4. 3D reconstruction of fluorescent FtsZ filaments imaged with a 3D SIM microscope of the fourth cell shown in Figure 2, related to Figure 2

We conclude that FtsZ can mediate cell division by polymerizing into a discontinuous square, implying that membrane curvature is not required for FtsZ function in a naturally occurring cuboid bacterium.

## Discussion

Although Polz et al. (1992) reported on corn-kernel shaped symbionts attached to the cuticle of *Catanema sp.* 1 from Croatia, no cell population thereof was subjected to rigorous morphometric analysis. Here, we showed that *Ca*. T. cuboideus cells are cuboid. Aside from the squared archaeal species, which were described as either flat (Oren, 1999) or only 0.25 μm thick (Stoeckenius, 1981), only two more examples of cuboid cells are known, both belonging to the eukaryotic kingdom of life: the first example are the cells that build the compound eyes (ommatidia) of decapod crustaceans (Palmer et al., 2018). The second example are cells that build the tightly sealed surface epithelia lining human organs (e.g., kidneys, ovaries). These, however, appeared as prisms when viewed from the top (Krstić, 1985). We hypothesize that, in an epithelium-like monolayer of *Ca.* T. cuboideus cells, a cuboid shape would maximize contact areas between neighboring cells more than a rod shape would. If, based on transcriptomics, *Ca.* T. oneisti might import phospholipids, ammonia, and organic compounds from its host and sulfide, oxygen, urea, and CO_2_ from the environment (Paredes et al., 2021), we still do not know which molecules Thiosymbion cells exchange among themselves. Therefore, the physiological needs that would drive the evolution of a cube-like cell await discovery.

We investigated the reproduction mode of a sulfur-oxidizing, cube-like gammaproteobacterium that naturally thrives on the surface of marine free-living nematodes. The proximal localization of fimbriae suggests that this symbiont uses these appendages to attach to its nematode host and that its growth and division may be host-polarized as shown for the phylogenetically related nematode symbiont, *Ca*. T. hypermnestrae (Leisch et al., 2016; Pende et al., 2018). It is possible that both symbionts start septal growth proximally, to guarantee host attachment to both daughter cells, even prior to septation completion.

The cytoskeleton is the major shape determinant for eukaryotic and prokaryotic cells. According to current models, in order to mediate bacterial septation, FtsZ polymerizes into a highly dynamic, discontinuous, and heterogeneous ring. In *E. coli*, this is facilitated by a multitude of proteins that (directly or indirectly) interact with FtsZ to form the so-called divisome (Egan et al., 2020; Typas et al., 2011). Analysis of the *Ca*. T. cuboideus genome draft let us identify all the divisome genes, except for the late divisome *ftsN*, *damX*, and *dedD* genes (Table S5). It has been suggested that cell pole morphogenesis in rod-shaped cells relies on FtsZ dynamics and structure, just like the controlled elongation of the lateral walls relies on the actin homolog MreB (McQuillen and Xiao, 2020). Although the *mreB* gene was identified in *Ca*. T. cuboideus, successful cultivation and genetic manipulation are necessary to track the localization pattern of fluorescently labeled FtsZ and MreB *in vivo* to understand their role in the morphogenesis of the cuboid symbiont.

In bacteria that were artificially forced to become cuboid, while their growth and division was pharmacologically halted, FtsZ polymerized into sharp-cornered Z-squares (Söderström et al., 2018). Even though in *Ca*. T. cuboideus we could not observe continuous Z-squares, we did detect sharp-cornered, staple-like FtsZ filaments (Figure 3 bottom panel, Video S4). This implies that FtsZ does not have to polymerize into half rings or arcs to mediate division and that membrane roundness is not required for FtsZ function in naturally occurring cells.

Even though only a handful of squared or cuboid shapes have been reported in prokaryotic and eukaryotic cells so far, this morphology might be more widespread than currently assumed. To know whether the natural occurrence of cube-like cells is truly that rare or whether they are simply under-sampled, we need to screen the Earth microbiome for morphological diversity by using high-throughput techniques. Only then we will have a more realistic picture of what is hiding behind rods and cocci.

### Limitations of the study

All thiotrophic symbionts, except for the one of the *K. polythalmia* shipworm (Distel et al., 2017), are, as of yet, uncultivable. Furthermore, filming bacteria attached to 1-cm-long nematodes is technically challenging mainly because of the high autofluorescence of the worm cuticle and because the filming should be carried out in anoxic conditions. Thus, we currently cannot visualize the localization pattern of cytoskeletal proteins *in vivo* or provide movies of live, nematode-attached *Ca.* T cuboideus.

## STAR★Methods

### Key resources table

**Table:** 

REAGENT or RESOURCE	SOURCE	IDENTIFIER
**Antibodies**		

Sheep polyclonal anti-*E. coli* FaeG	Abcam	Abcam Cat#ab35292; RRID: AB_732234
Rabbit polyclonal anti-*E. coli* FtsZ	Agrisera	AgriSera Cat#AS10 715; RRID: AB_10754647

**Chemicals, peptides, and recombinant proteins**		

Wheat germ agglutinin fluorescein conjugate	ThermoFisher	Cat#W834
EDA-DA	VanNieuwenhze, Indiana University Bloomington	N/A

**Critical commercial assays**		

Click-iT EdU Alexa Fluor 488 Imaging Kit	Invitrogen	Cat#C10337
Ligation sequencing kit	Oxford Nanopore Technologies	Cat#SQK-LSK109
R9.4 flow cell	Oxford Nanopore Technologies	Cat#FLO-Min106

**Deposited data**		

Genome draft for *Candidatus* Thiosymbion cuboideus	This paper	GenBank: WYCW00000000.1
*Candidatus* Thiosymbion cuboideus raw sequencing reads	This paper	SRA: SRR13336336
Symbiont of *Eubostrichus fertilis ftsZ* sequence	This paper	GenBank: OL343678
Symbiont of *Eubostrichus dianeae ftsZ* sequence	This paper	GenBank: OL343677

**Experimental models: Organisms/strains**		

*Candidatus* Thiosymbion cuboideus	Environmental sample collected by the authors for this paper	N/A

**Software and algorithms**		

ImageJ	Schneider et al. (2012)	https://imagej.nih.gov/ij/
Fiji	Schindelin et al. (2012)	https://imagej.net/Fiji
ObjectJ	van der Ploeg et al. (2013); Vischer et al. (2015)	https://sils.fnwi.uva.nl/bcb/objectj/
ProgRes Capture Pro 2.8.8	Jenoptik	https://www.jenoptik.us/
Photoshop CC/2021	Adobe Systems	https://www.adobe.com/
Illustrator CC/2021	Adobe Systems	https://www.adobe.com/
Indesign CC/2021	Adobe Systems	https://www.adobe.com/
SoftWoRx	GE Healthcare Life Sciences	https://www.bioz.com
Guppy Basecalling Software	Oxford Nanopore Technologies	https://nanoporetech.com
Porechop	Ryan Wick	https://github.com/rrwick/Porechop
bbduk	N/A	https://sourceforge.net/projects/bbmap/
Unicycler	Wick et al., (2017)	https://github.com/rrwick/Unicycler
mmgenome2	Kasper Skytte Andersen, Rasmus Kirkegaard	https://github.com/KasperSkytte/mmgenome2
SSPACE LongRead	Boetzer and Pirovano (2014)	https://github.com/Runsheng/sspace_longread
Gapfiller.pl	Nadalin et al. (2012)	https://sourceforge.net/projects/gapfiller/
Pilon	Walker et al. (2014)	https://github.com/broadinstitute/pilon
checkM	Parks et al. (2015)	https://github.com/Ecogenomics/CheckM
Prokka	Seemann (2014)	https://github.com/tseemann/prokka
mafft	Katoh and Standley (2013)	https://mafft.cbrc.jp/alignment/software/
TrimAl	Capella-Gutiérrez et al. (2009)	https://bio.tools/trimal
IQ-TREE	Minh et al. (2020)	http://www.iqtree.org/
ModelFinder Plus	Kalyaanamoorthy et al. (2017)	http://www.iqtree.org/
FigTree	N/A	http://tree.bio.ed.ac.uk/software/figtree/
RStudio	N/A	http://www.R-project.org/
Ggmsa (R)	N/A	http://yulab-smu.top/ggmsa/
blastp	Altschul et al. (1990)	N/A
heatmap.2 (R)	N/A	http://www.R-project.org/
prodigal	Hyatt et al., (2010)	https://github.com/hyattpd/Prodigal
pfam	Mistry et al., (2021)	http://pfam.xfam.org/

### Resource availability

#### Lead contact

• Further information and requests for resources and reagents should be directed to and will be fulfilled by the lead contact, Silvia Bulgheresi (silvia.bulgheresi@univie.ac.at).

#### Materials availability

• This study did not generate new strains or unique reagents.

### Experimental model and subject details

*Catanema sp.* “Guadeloupe” individuals were collected in 2018 from sand bars in Guadeloupe, French West Indies (Ilet à Cochons, 16°12′53.76″N 61°32′05.74″W) at approximately 1 m depth. The sand was collected with the aid of cores and nematodes were extracted by gently stirring the sand in seawater and subsequently pouring it onto a 212 μm mesh sieve. Single adult worms of both sexes (1-2 mm length) were manually picked (Dumont 3, Fine Science Tools, Canada) under a dissecting microscope. *Catanema sp.* nematodes were identified based on morphological characteristics (Scharhauser et al., 2020).

### Method details

#### Transmission electron microscopy

Live worms were plunge frozen in liquid propane at −179°C, cryo-substituted rapidly in acetone using the agitation module described in Goldammer et al. (2016) and transferred in absolute ethanol. Dehydrated samples were then embedded in medium-grade LR White resin. Polymerization was performed under nitrogen atmosphere at 40°C for three days. Alternatively, live worms were chemically fixed in a modified Trump’s fixative solution (2.5% glutaraldehyde, 2% paraformaldehyde in sodium cacodylate 0.1 mol L^−1^; 1,000 mOsm L^−1^; pH 7.2; Trump and Ericsson, 1965) and further dehydrated in ascending ethanol series before embedding in Agar Low Viscosity Resin (Agar Scientific®). Thin sections (70 nm) were placed on Formvar®-coated slot grids and stained with 0.5% uranyl acetate and 3% lead citrate prior to imaging with a Zeiss® Libra 120 transmission electron microscope.

#### Scanning electron microscopy

Whole worms were fixed in modified Trump’s fixative solution (2.5% glutaraldehyde and 2% paraformaldehyde in 0.1 M sodium cacodylate buffer, 1,000 mOsm L^−1^, pH 7.2) (Trump and Ericsson, 1965). To dissociate the bacteria, 10 nematodes were washed three times by pipetting them up and down in 100 μL of 0.1 M sodium cacodylate buffer. 50 μl of the bacterial suspension was spotted on a poly-L-lysine-coated glass slide and let sink for 15 min for proper attachment. The samples were then dehydrated in an ascending ethanol series, followed by 100 % acetone, and critical-point drying (Leica EM CPD300, Leica Microsystems, Wetzlar, Germany). Finally, they were mounted on stubs and sputter-coated with gold (JEOL JFC-2300HR, Tokyo, Japan), and observed on an IT 300 scanning electron microscope (JEOL).

#### DNA extraction, sequencing and genome assembly of *Ca.* T. cuboideus

500 *Catanema* sp. “Guadeloupe” nematodes collected in 2018 were used for DNA extraction and genome assembly of their ectosymbiont. The bacteria were detached from the worms by dipping the nematodes into ddH2O for 1 min, then transferring them to 0.2 μM-filtered seawater for 5 min, after which the symbiont-free nematodes were discarded, and the bacterial suspensions pooled and collected by centrifugation. The bacterial fraction was carefully resuspended in TLB (100 mM NaCl, 10 mM Tris-HCl pH 8, 25 mM EDTA pH 8, 0.5% v/v SDS), 10 μL RNase A (20 mg/ml, Thermo Fisher) and 10 μL lysozyme in lysozyme buffer (100 mg/ml in 20 mM Tris-HCl pH 8, 2 mM EDTA pH 8.0, 1% v/v Triton X-100) were added, mixed by inverting the tube and incubated at 37°C. After 1 h, 30 μL Proteinase K (20 mg/ml) was added, the tube inverted and incubated for 50°C for 1 h. For the phenol-chloroform extraction, the lysate was mixed with 500 μL Phenol:Chloroform:Isoamyl alcohol (25:24:1, v/v Thermo Fisher Scientific), vortexed for 1 min and centrifuged for 5 min at 4°C and 16 000 x g. The aqueous phase was transferred to a new tube, 500 μL Chloroform:Isoamyl alcohol (24:1) added, vortexed for 1 min and again centrifuged. The aqueous phase was then mixed with 0.3 volumes 7.5 M NH4OAc (pH 5.2), 20 μg glycogen and 2 volumes of ice-cold ethanol 100%. The solution was incubated for 15 min at room temperature and the DNA subsequently pelleted by centrifugation for 30 min at 4°C and 16 000 x g. The supernatant was carefully taken off, and the DNA pellet washed with 80% ethanol. The supernatant was completely taken off, and the DNA pellet allowed to air dry for 5 min. DNA was resuspended in 20 μL of PCR molecular grade water for 1 h at 37°C.

The library for Oxford Nanopore Technologies (ONT) sequencing was prepared using the ONT 1D ligation sequencing kit (SQK-LSK109) and sequenced on a R9.4 flow cell (FLO- MIN106) on a MinION for 48 h. Base calling was performed locally with ONT’s Guppy Basecalling Software v3.2.4+d9ed22f, and resulting fastq-files were trimmed using Porechop version 0.2.1 (https://github.com/rrwick/Porechop).

For the assembly, raw Illumina reads were quality filtered and trimmed using bbduk version 37.61 (https://sourceforge.net/projects/bbmap/) by a minimum quality value of 2 and minimum length of 50. Unicycler version 0.4.6 (Wick et al., 2017) was used in ‘bold’ mode to assemble the trimmed Illumina and ONT reads, with SPAdes version 3.13.1 in ‘careful mode. The assembly was manually binned using the mmgenome2 tool (https://github.com/KasperSkytte/mmgenome2). The bin was further scaffolded using SSPACE LongRead version 1.1 (Boetzer and Pirovano, 2014) with the trimmed ONT reads, and ambiguous bases replaced using Gapfiller.pl version 1.10 (Nadalin et al., 2012), followed by 10 rounds of Pilon version 1.22 (Walker et al., 2014) using BWA-aln mapped reads, and contigs shorter than 200 bp were discarded.

The genome completeness was assessed using checkM version 1.0.18 (Parks et al., 2015) with the gammaproteobacterial marker gene set using the taxonomy workflow. Assembled contigs were annotated using Prokka 1.14..6 (Seemann, 2014). The genome sequence has been deposited at GenBank under the accession WYCW00000000.1. Raw reads, basecalled with Guppy Basecalling Software 4.2.2+effbaf8, have been deposited in SRA under the accession SRR13336336.

#### Symbiont phylogeny, FtsZ sequence alignment and annotation of divisome proteins

For the 16S phylogenetic analysis, 48 *Ca*. Thiosymbion 16S sequences from Scharhauser et al., (2020) were retrieved from GenBank together with 16S sequences from 3 outgroup species (see Table S1 for accession numbers). The alignment was performed with mafft v7.427 (L-INS-I mode, Katoh and Standley, 2013), trimmed in TrimAl v1.4.rev15 (-gt 0.7, Capella-Gutiérrez et al., 2009) and the maximum likelihood phylogeny reconstructed using IQ-TREE v2.1.2 (Minh et al., 2020) with the best-fit model automatically selected by ModelFinder Plus (Kalyaanamoorthy et al., 2017) and 1,000 ultrafast bootstraps (Hoang et al., 2018). The phylogeny was outgroup-rooted and visualized in FigTree v1.4.4 (http://tree.bio.ed.ac.uk/software/figtree/).

Six gammaproteobacterial FtsZ sequences (Table S2) were aligned using mafft and visualized with ggmsa in R (http://www.R-project.org/). Identities were retrieved by a pairwise blastp (Altschul et al., 1990) and plotted with heatmap.2 in R (http://www.R-project.org/).

Divisome proteins were annotated by predicting protein-coding genes using prodigal v2.6.3 (Hyatt et al., 2010) on the *Ca*. T. cuboideus genome draft (WYCW00000000.1) and blasted against Swiss-Prot *E. coli* K12 proteins using blastp v2.11.0 of the NCBI blast+ software suite (https://pubmed.ncbi.nlm.nih.gov/20003500/), and reverse blasted against the full *E. coli* K12 proteome. Domain architecture was verified using the pfam online server against Pfam 34 (Mistry et al., 2021). Divisome proteins, their role and categories were retrieved from Egan et al. (2020) and Typas et al. (2011).

#### Wheat germ agglutinin staining

Deep-frozen methanol-fixed nematodes were rehydrated and washed in PBS, followed by incubation in 20 μg/mL FITC labelled wheat germ agglutinin (WGA; W834, Invitrogen) in PBS for 1h at room temperature. Unbound WGA was removed by three washing steps in PBS and worms were sonicated for 40 s to dissociate *Ca*. T. cuboideus cells from their hosts prior mounting. 1 μl of the bacterial solution was mixed with 0.5 μl of Vectashield mounting medium (Vector Labs).

#### Cell size and fluorescence measurements

Symbiont cells were dissociated from fixed *Catanema sp*. “Guadeloupe” nematodes by sonication. Cell suspensions were applied to an 1% agarose covered microscopy slide and imaged using a Nikon Eclipse Ni microscope equipped with a MFCool camera (Jenoptik). Epifluorescence images were acquired using ProgRes Capture Pro 2.8.8 software (Jenoptik) and processed using the public domain program ImageJ (Schneider et al., 2012) in combination with plugin ObjectJ and a modified version of Coli-Inspector (van der Ploeg et al., 2013; Vischer et al., 2015). Cell length, width and fluorescence patterns were measured automatically. Automatic cell recognition was double-checked manually. For the average fluorescence plots and for the demographs, cells were grouped into morphological classes, each cell was resampled to the same length or width and the fluorescence intensities added up and averaged. For representative images, the background subtraction function of ImageJ was used and brightness and contrast were adjusted for better visibility. Data analysis was performed using Excel 365 (Microsoft Corporation, USA), plots were created with R Studio (1.4.1103) and figures were compiled using Illustrator CC (Adobe Systems Inc. USA).

#### EDA-DA incubation of live symbionts

To track symbiont cell wall growth, batches of approx. 50 live symbiotic *Catanema sp*. “Guadeloupe” nematodes were each incubated in a 1.5 mL tube containing 500 μl of 10 mM ethynyl-D-alanyl-D-alanine (EDA-DA, a D-amino acid carrying a clickable ethynyl group; synthetized by Michael S. VanNieuwenhze, Indiana University) in filter sterilized natural seawater (FSW) for 180 min and subsequently washed once in FSW, transferred to methanol, and stored at −20°C. Nematodes were transported from Guadeloupe to Vienna deep-frozen.

#### Click-chemistry

EDA-DA incubated, deep frozen methanol-fixed *Catanema sp*. “Guadeloupe” nematodes were rehydrated and washed in PBS containing 0.1% Tween 20 (PBT). Blocking was carried out for 30 min in PBS containing 0.1% Tween 20 (OBT) and 2% (wt/vol) bovine serum albumin (blocking solution) at room temperature. An Alexa488 fluorophore was covalently bound to EDA-DA via copper catalyzed click-chemistry by following the user manual protocol for the Click-iT reaction cocktail (Click-iT EdU Imaging Kit, Invitrogen). The nematodes were incubated with the Click-iT reaction cocktail for 30 min at RT in the dark. Unbound dye was removed by a 10-min wash in PBT and one wash in PBS. Worms were sonicated for 40 s to dissociate *Ca*. T. cuboideus prior mounting.

#### Immunostaining

Deep-frozen methanol-fixed *Catanema sp*. “Guadeloupe” nematodes were rehydrated and washed in PBT, followed by blocking for 1 h in PBT containing 2% (wt/vol) bovine serum albumin (blocking solution) at room temperature. *Ca*. T. cuboideus were incubated with a 1:200 dilution of commercially available rabbit polyclonal anti-*E. coli* FtsZ antibody (Agrisera) in blocking solution, as well as with a 1:500 dilution of sheep polyclonal anti-*E. coli* K88 fimbrial protein AB/FaeG antibody (ab35292, Abcam). Both the anti-*E. coli* FtsZ antibody (Agrisera) and the anti-*E. coli* K88 fimbrial protein AB/FaeG antibody (ab35292, Abcam) were previously shown to specifically recognize the FtsZ and the fimbriae of other Thiosymbion ectosymbionts (Leisch et al., 2012; Pende et al., 2018). All primary antibodies were incubated overnight at 4°C in blocking solution. Upon incubation with primary antibody (or without in the case of the negative control) samples were washed three times in PBT and incubated with secondary Alexa488 conjugated anti-rabbit (Jackson ImmunoResearch) for anti-*E. coli* FtsZ antibody and Alexa555 conjugated anti-sheep antibody (Thermo Fisher Scientific) for anti-*E. coli* K88 fimbrial AB/FaeG antibody, both at 1:500 dilution in blocking solution for 1 h at room temperature. Unbound secondary antibody was removed by three washing steps in PBT and worms were sonicated for 40 s to dissociate *Ca*. T. cuboideus cells from their hosts prior mounting. 1 μL of the bacterial solution was mixed with 0.5 μL of Vectashield mounting medium (Vector Labs).

#### Three-dimensional structured illumination microscopy (3D SIM) imaging and analysis

Symbiont cell suspensions were applied on high precision coverslips (No. 1.5H, Sigma-Aldrich) coated with 0.01% (wt/vol) of Poly-L-Lysin. After letting the cell dry onto the surface of the coverslip, antifade mounting medium (Vectashield) was applied and the coverslip was sealed to a slide. 3D SIM was performed on a Delta Vision OMX v4 microscope equipped with an Olympus 60X/1.42 Oil Plan Apo N objective or an Olympus 100X/1.42 Oil Plan Apo N objective and 2 sCMOS or EMCCD cameras. The samples were excited with lasers at 488 nm, the emission was detected through emission filters 477/32 nm (Center/Bandpass). The image reconstruction and registration were performed using the SoftWoRx image software running under Linux operating system. For further image analysis of SIM image z stacks, we used Fiji (Schindelin et al., 2012) Version 2.0.0-rc-54/1.51i. Namely, brightness and contrast were adjusted, stacks were fused to a single image (z projection, maximum intensity), stacks were rotated 90° (resliced) prior z projection for the 90° side view, and videos were created via 3D projection. Regions of interest were cut out and, for uniformity, placed on a black squared background. For clarity a black triangle was placed in the corner of the lower cell in Figure 1C to cover the fluorescence of a neighboring cell. Figures were compiled using Illustrator CC (Adobe Systems Inc. USA).

### Quantification and statistical analysis

Microscopic images were processed using the public domain program ImageJ (Schneider et al., 2012) in combination with plugin ObjectJ (Vischer et al., 2015) or Fiji (Schindelin et al., 2012). For the 2D morphometry, cell axes were traced, and morphometric measurements recorded. Automatic cell recognition was double-checked manually. Normalized fluorescence intensities were plotted against the normalized cell width. For the demographs, each cell was resampled to the same length and the fluorescence intensities added up and averaged. Data analysis was performed using Excel for Mac, plots were created with Excel and RStudio (https://www.r-project.org/) and figures were compiled using Illustrator 2021 and Indesign 2021.

## Data Availability

• The symbiont genome draft has been deposited at DDBJ/ENA/GenBank under the accession number GenBank: WYCW00000000.1 and has been publicly available as of the date of publication. Raw sequencing reads have been deposited in SRA under the accession SRA: SRR13336336 publicly available as of the date of publication. All further data reported in this paper will be shared by the lead contact upon request. Accession numbers are listed in the key resources table. • This paper does not report original code. • Any additional information required to reanalyze the data reported in this paper is available from the lead contact upon request.
