# Supplementary material for: FtsZ-mediated fission of a cuboid bacterial symbiont
Source: iScience. 2021 Dec 2;25(1):103552. doi: 10.1016/j.isci.2021.103552 (PMC8760462; doi:10.1016/j.isci.2021.103552)
Supplement: Document S1. Figures S1–S3 and Tables S1–S5 [file mmc1.pdf]

iScience, Volume *25*

Supplemental information

FtsZ-mediated fission

# of a cuboid bacterial symbiont

Philipp M. Weber, Gabriela F. Paredes, Tobias Viehboeck, Nika Pende, Jean-Marie Volland, Olivier Gros, Michael VanNieuwenhze, Jörg Ott, and Silvia Bulgheresi

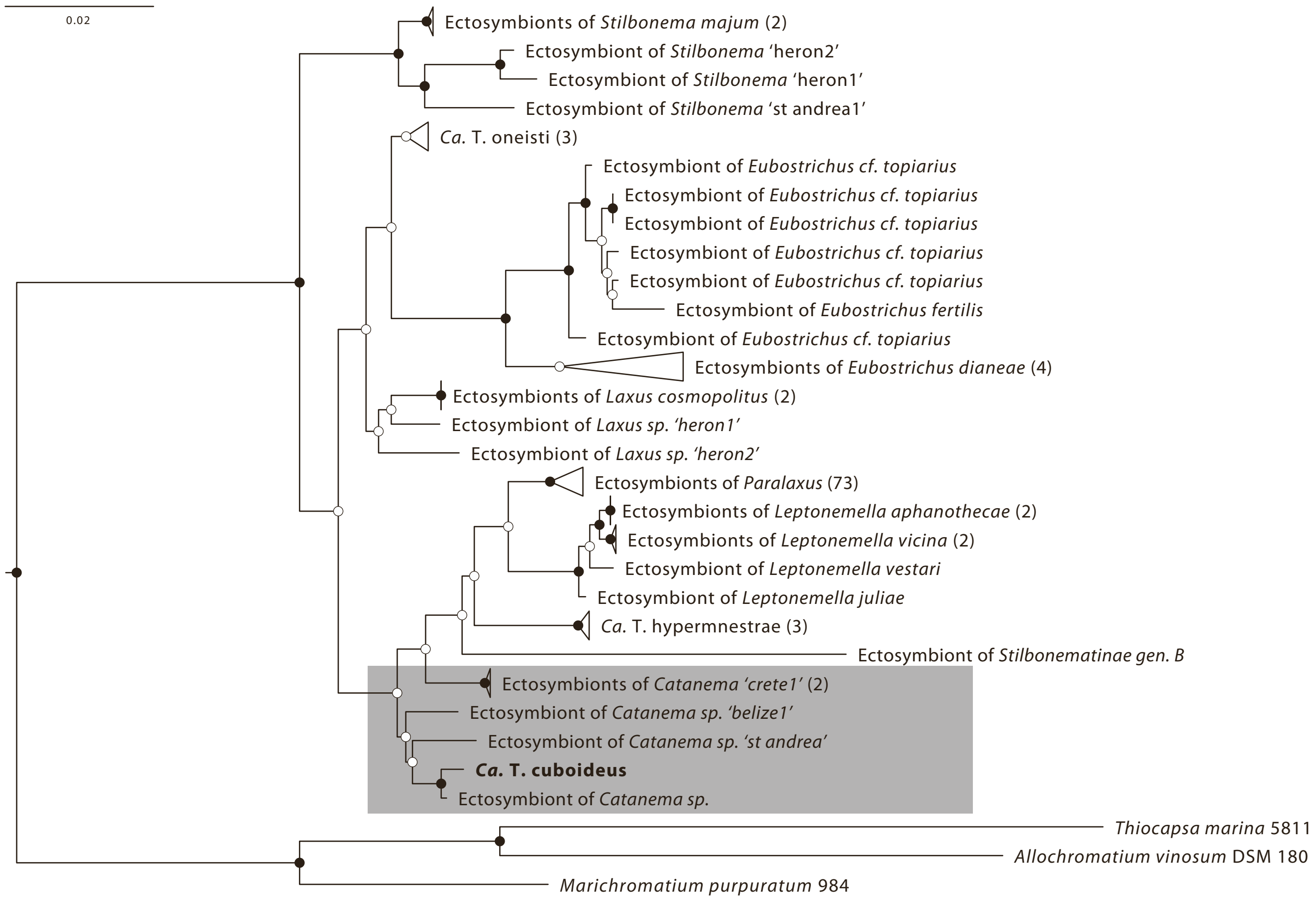

Figure S1. 16S rRNA gene-based phylogenetic placement of *Ca.* T. cuboideus. Related to Figure 1. Maximum-likelihood reconstruction of 48 16S rRNA-genes of marine nematodes (*Stilbonematinae*) ectosymbionts. The tree was rooted using *Allochromatium vinosum* DSM 180, *Marichromatium purpuratum* 984 and *Thiocapsa marina* 5811 as an outgroup. Black circles indicate ultrafast bootstrap support values of ≥ 95%, all other node support values (<95%) are depicted by open circles. For better readability, some branches have been collapsed and number of sequences is noted in brackets. See Table S2 for a list of GenBank accession numbers. See also Table S2.

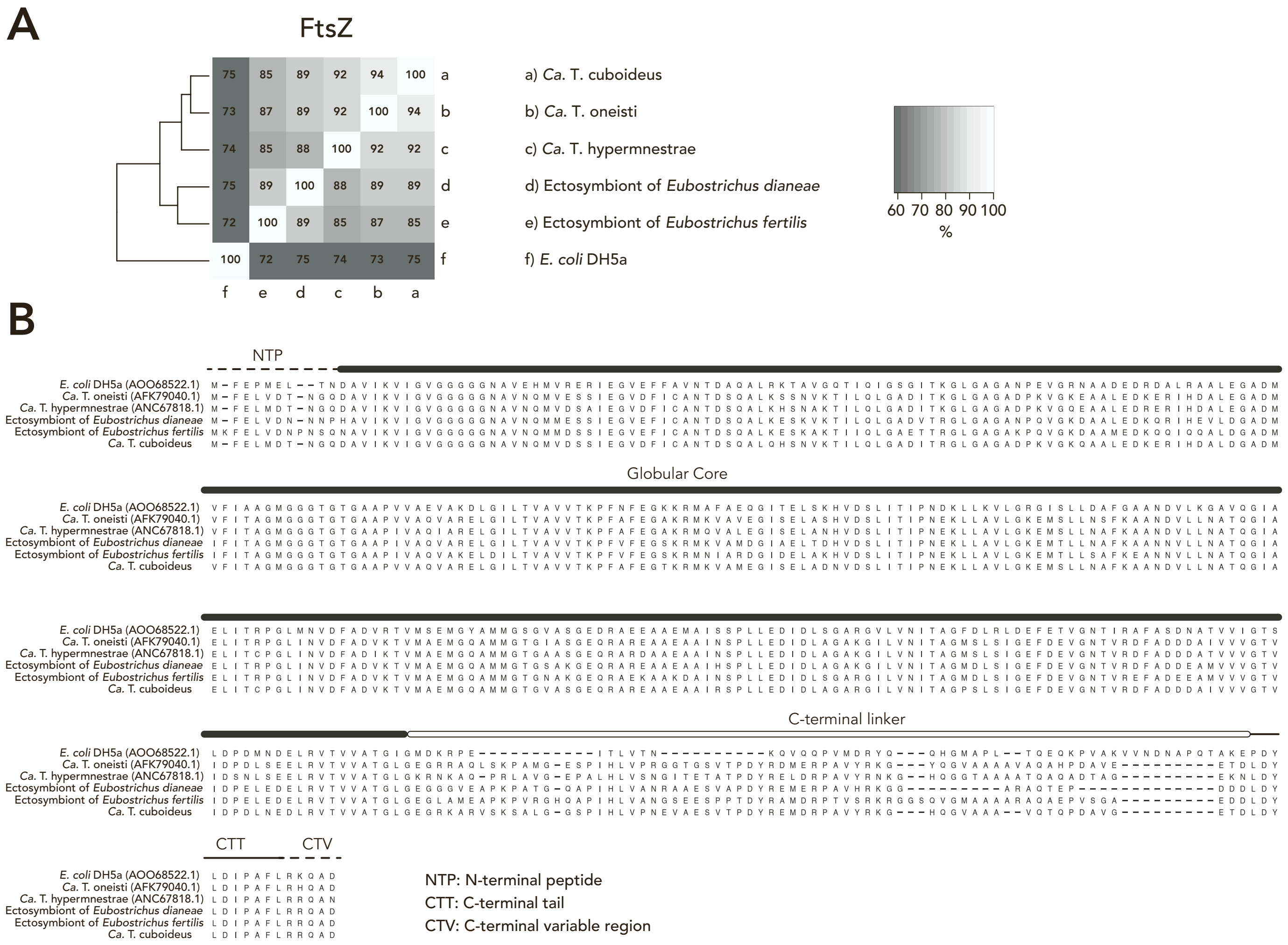

Figure S2. FtsZ identity heatmap and FtsZ amino acid sequence alignment. Related to Figures 3 and 4. (A and B) Heatmap and alignment of FtsZ sequences of *Escherichia coli* DH5α, *Ca.* T. oneisti, *Ca.* T. cuboideus, and of ectosymbionts of *Eubostrichus dianeae* and *E. fertilis*. (A) Heatmap showing the identity of pairwise-aligned FtsZ sequences in percentage. Sequences are clustered via hierarchical clustering (complete linkage method) using Euclidean distances. (B) In the alignment five distinct functional regions of FtsZ are indicated, N-terminal peptide (NTP, dashed line), Globular Core (thick black line), C-terminal linker (thick white line), C-terminal tail (CTT, thin black line) and C-terminal variable region (CTV, dashed line end). Functional regions based on Casiraghi et al., 2020 and Silber et al., 2020. See also Table S4.

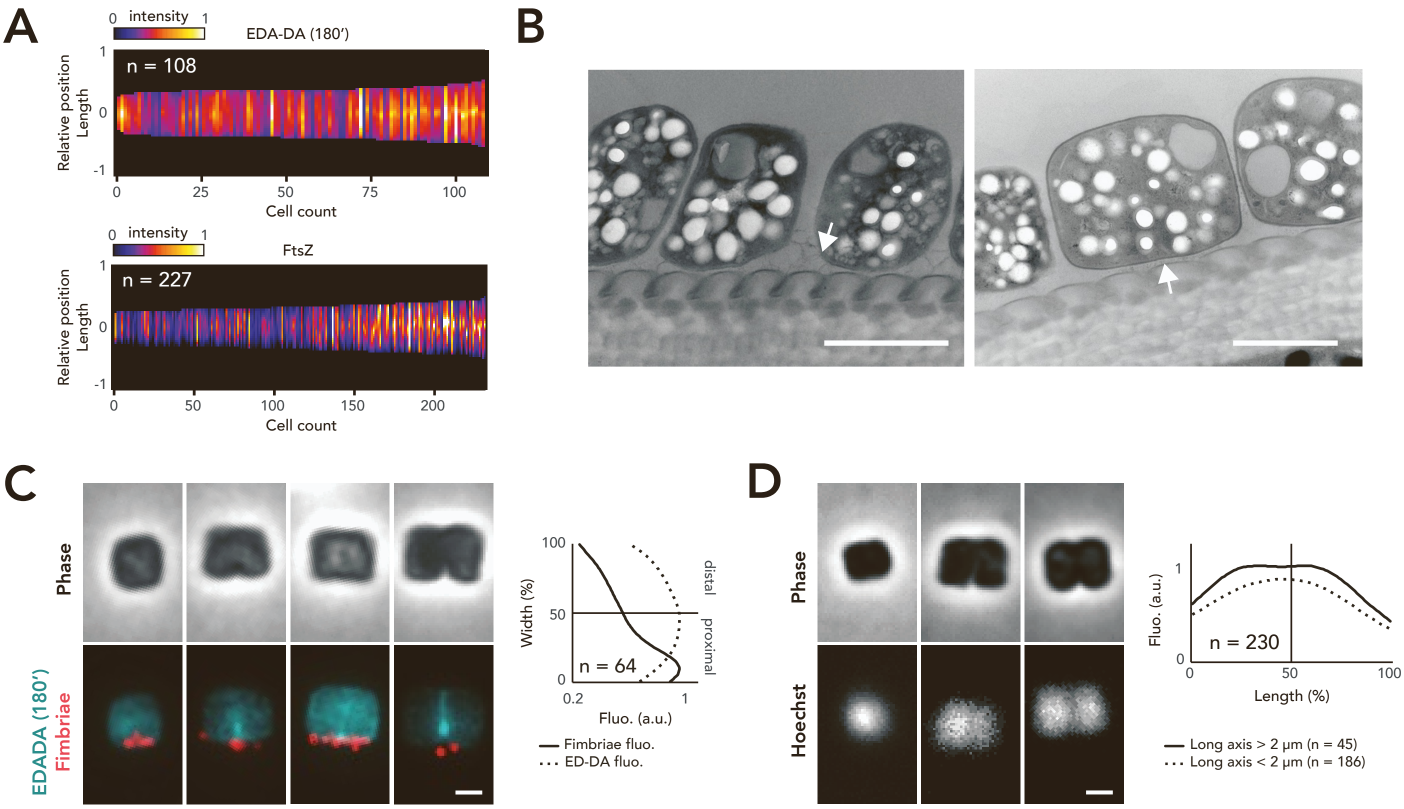

Figure S3. Localization patterns of EDA-DA, FtsZ, fimbriae and DNA, as well as transmission electron micrographs (TEM) of *Ca.* T. cuboideus. Related to Figures 1 and 3.
(A) Demographs of *Ca.* T. cuboideus cells labeled with a PG metabolic probe (EDA-DA) for 180 minutes (top, n = 108) and immunostained with anti-FtsZ antibody (bottom). Each cell is represented as a pixel-wide bar representing the Length axis. The cells were sorted according to increasing length from left to right. Of note, Length and Depth axis are indistinguishable in non-dividing cells. (B) TEM images display fimbrial structures between the symbiont cell and the host cuticle (white arrowheads). (C) Epifluorescence images of four representative *Ca.* T. cuboideus cells, sorted by increasing length from left to right, incubated with EDA-DA and immunostained with an anti-fimbriae antibody. Plot shows normalized fluorescence emitted by 62 *Ca.* T. cuboideus cells (a.u.) plotted against their cell width (%). (D) Representative symbiont cells stained with the DNA stain Hoechst and sorted from the youngest to the oldest. Plot shows the total fluorescence (a.u.) emitted by short cells (long axis <2 µm; full line; n = 45) and long cells (long axis >2 µm; dotted linen = 186) plotted against their length (%). Scale bars corrspond to 1 µm.

**Genome size [Mb]**	5.006632
**Number of contigs**	16
**Average G+C content [%]**	57.51
**Protein coding density [%]**	82.67
**Number of coding sequences (CDS)**	6316
**Number of tRNA**	45
**Number of rRNA**	3 (1 operon)
**CheckM Completeness [%]**	96.61
**CheckM Contamination [%]**	2.28
**CheckM Strain heterogeneity [%]**	16.67

Table S1. Related to Figure 1. *Ca.* T. cuboideus genome features.

**Organism**	**GeneBank**
Ca. T. hypermnestrae	KP943980.1
Ca. T. hypermnestrae	EU711428.1
Ca. T. hypermnestrae	LR746256.1
Ca. T. oneisti	LR746261.1
Ca. T. oneisti	KT826595.1
Ca. T. oneisti	KF278591.1
Ectosymbiont of *Catanema 'crete1'*	LR746257.1
Ectosymbiont of *Catanema 'crete1'*	LR746258.1
Ectosymbiont of *Catanema sp.*	EU711426.1
Ectosymbiont of *Catanema sp. 'belize1'*	KP943972.1
Ectosymbiont of *Catanema sp. 'st andrea'*	KP943973.1
Ectosymbiont of *Eubostrichus cf. dianeae*	KP943975.1
Ectosymbiont of *Eubostrichus cf. topiarius*	LR746246.1
Ectosymbiont of *Eubostrichus cf. topiarius*	LR746244.1
Ectosymbiont of *Eubostrichus cf. topiarius*	LR746245.1
Ectosymbiont of *Eubostrichus cf. topiarius*	LR746242.1
Ectosymbiont of *Eubostrichus cf. topiarius*	LR746243.1
Ectosymbiont of *Eubostrichus cf. topiarius*	KP943974.1
Ectosymbiont of *Eubostrichus dianeae*	LR746262.1
Ectosymbiont of *Eubostrichus dianeae*	LR746247.1
Ectosymbiont of *Eubostrichus dianeae*	KF278587.1
Ectosymbiont of *Eubostrichus fertilis*	KF278590.1
Ectosymbiont of *Laxus cf. cosmopolitus*	EU711427.1
Ectosymbiont of *Laxus cf. cosmopolitus*	KP943986.1
Ectosymbiont of *Laxus cf. cosmopolitus*	LR746259.1
Ectosymbiont of *Laxus sp. 'heron1'*	KP943987.1
Ectosymbiont of *Laxus sp. 'heron2'*	KP943988.1
Ectosymbiont of *Leptonemella aphanothecae*	KP943979.1
Ectosymbiont of *Leptonemella aphanothecae*	LR746248.1
Ectosymbiont of *Leptonemella juliae*	KP943977.1
Ectosymbiont of *Leptonemella vestari*	KP943976.1
Ectosymbiont of *Leptonemella vicina*	KP943978.1
Ectosymbiont of *Leptonemella vicina*	KU921521.1
Ectosymbiont of *Paralaxus 'heron1'*	KP943985.1

Ectosymbiont of *Paralaxus 'oahu1'*	LR746253.1
Ectosymbiont of *Paralaxus 'oahu1'*	LR746252.1
Ectosymbiont of *Paralaxus bermudensis*	LR746255.1
Ectosymbiont of *Paralaxus bermudensis*	LR746250.1
Ectosymbiont of *Paralaxus bermudensis*	LR746249.1
Ectosymbiont of *Paralaxus cocos*	LR746254.1
Ectosymbiont of *Paralaxus cocos*	KP943984.1
Ectosymbiont of *Stilbonema 'heron1'*	KP943983.1
Ectosymbiont of *Stilbonema 'heron2'*	KP943982.1
Ectosymbiont of *Stilbonema 'st andrea1'*	KP943981.1
Ectosymbiont of *Stilbonema majum*	LR746260.1
Ectosymbiont of *Stilbonema majum*	HM776017.1
Ectosymbiont of *Stilbonematinae gen. B*	KP943971.1
Ectosymbiont of *Paralaxus 'heron1'*	LR746251.1
Allochromatium vinosum DSM 180	CP001896.112452.113967
Marichromatium purpuratum 984	CP007031.294591.296118
Thiocapsa marina 5811	AF112998.1
Ca. T. cuboideus	WYCW01000004.1.400935.402467

Table S2. Related to Figure S1. 16S rRNA-gene sequences used for the phylogenetic reconstruction displayed in Figure S1 (from Scharhauser et al. 2020).

Number of cells (n)		Length (µm)	Width (µm)
All (256)	average	2.01	1.70
	stdev	0.45	0.11
Non-dividing (212)	average	1.87	1.71
	stdev	0.32	0.14
Dividing (44)	average	2.70	1.64
	stdev	0.31	0.14

Table S3. Related to Figure 2. Phase contrast image-based morphometry of dissociated *Ca.* T. cuboideus cells. Of note, Length and Depth axis are indistinguishable in non-dividing cells.

Organism	GeneBank
E. coli DH5a	AOO68522.1
Ca. T. oneisti	AFK79040.1
Ca. T. hypermnestrae	ANC67818.1
Ectosymbiont of *Eubostrichus dianeae*	GenBank: OL343677
Ectosymbiont of *Eubostrichus fertilis*	GenBank: OL343678
Ca. T. cuboideus	WYCW01000003.1.112454-113641(-)

Table S4. Related to Figure 3. FtsZ amino acid sequences used for the heatmap and the alignment in Figure S2. See also Figure S2.

Activity or category	Protein	Role	*Ca.* T. cuboideus (WYCW00000000.1) open reading frames
Cytoskeletal structure, GT-Pase	FtsZ	Master regulator of cell division	WYCW01000003.1.112454-113641(-)
'Early' association with the Z ring	FtsA	Stabilization and membrane-attachment of FtsZ polymers	WYCW01000003.1.113759-114994(-)
	ZipA		WYCW01000002.1.178641-179393(+)
	ZapA		WYCW01000001.1544827-1545129(-)
	ZapB		N/D
	ZapC		N/D
	FtsE	Regulate FtsA dynamics	WYCW01000003.1.234086-234751(-)
	FtsX		WYCW01000003.1.232406-233377(-)
'Late' association with the Z ring	FtsK	Recruitment of proteins and DNA transport	WYCW01000002.1.312104-314440(-)
	FtsQ	Interactions with peptidoglycan synthases	WYCW01000003.1.115000-115857(-)
	FtsL		WYCW01000003.1.136075-136350(-)
	FtsB		WYCW01000002.1.150096-150422(+)
	FtsW	Lipid II flippase	WYCW01000003.1.123198-124523(-)
	FtsI (PBP-3)	Peptidoglycan crosslinking during division	WYCW01000003.1.136075-136350(-)
	MurJ	Lipid II transport across the membrane	WYCW01000001.1.1096885-1098426(-)
	FtsN	Peptidoglycan binding	N/D
	DamX		N/D
	DedD		N/D
	RlpA		WYCW01000005.1.122818-123705(+)
Outer membrane invagination	TolQ	Outer membrane constriction during cell division, coordination with peptidoglycan synthesis	WYCW01000001.1754786-1755475(-)
	TolR		WYCW01000001.1754090-1754599(-)
	TolA		WYCW01000001.752474-1753469(-)
	TolB		WYCW01000001.1748660-1749955(-)
	Pal		WYCW01000001.1747915-1748496(-)

Table S5. Related to Figures 3 and 4. Presence and absence of bacterial divisome genes in the *Ca.* T. cuboideus genome draft. Based upon Egan et al., 2020 and Typas et al., 2011. N/D: not detected.
